# Comparative Genome Analysis of *Lactobacillus rhamnosus* Clinical Isolates from Initial Stages of Dental Pulp Infection: Identification of a New Exopolysaccharide Cluster

**DOI:** 10.1371/journal.pone.0090643

**Published:** 2014-03-14

**Authors:** Mangala A. Nadkarni, Zhiliang Chen, Marc R. Wilkins, Neil Hunter

**Affiliations:** 1 Institute of Dental Research, Westmead Centre for Oral Health and Westmead Millennium Institute, Westmead, New South Wales, Australia; 2 Faculty of Dentistry, The University of Sydney, Sydney, New South Wales, Australia; 3 Systems Biology Initiative, School of Biotechnology and Biomolecular Sciences, The University of New South Wales, Sydney, New South Wales, Australia; Université d'Auvergne Clermont 1, France

## Abstract

The human oral microbiome has a major role in oral diseases including dental caries. Our studies on progression of caries infection through dentin and more recently, the invasion of vital dental pulp, detected *Lactobacillus rhamnosus* in the initial stages of infection of vital pulp tissue. In this study employing current high-throughput next generation sequencing technology we sought to obtain insight into genomic traits of tissue invasive *L. rhamnosus*, to recognise biomarkers that could provide an understanding of pathogenic potential of lactobacilli, generally regarded as safe. Roche GS FLX+ technology was used to generate whole genome sequences of two clinical isolates of *L. rhamnosus* infecting vital pulp. Detailed genome-wide comparison of the genetic profiles of tissue invasive *L. rhamnosus* with probiotic *L. rhamnosus* was performed to test the hypothesis that specific strains of *L. rhamnosus* possessing a unique gene complement are selected for the capacity to invade vital pulp tissue. Analysis identified 264 and 258 genes respectively, from dental pulp-invasive *L. rhamnosus* strains LRHMDP2 and LRHMDP3 isolated from two different subjects that were not present in the reference probiotic *L. rhamnosus* strain ATCC 53103 (GG). Distinct genome signatures identified included the presence of a modified exopolysaccharide cluster, a characteristic confirmed in a further six clinical isolates. Additional features of LRHMDP2 and LRHMDP3 were altered transcriptional regulators from RpoN, NtrC, MutR, ArsR and zinc-binding Cro/CI families, as well as changes in the two-component sensor kinase response regulator and ABC transporters for ferric iron. Both clinical isolates of *L. rhamnosus* contained a single *Spa*FED cluster, as in *L. rhamnosus* Lc705, instead of the two *Spa* clusters (*Spa*CBA and *Spa*FED) identified in *L. rhamnosus* ATCC 53103 (GG). Genomic distance analysis and SNP divergence confirmed a close relationship of the clinical isolates but segregation from the reference probiotic *L. rhamnosus* strain ATCC 53103 (GG).

## Introduction

The human oral microbiome has a major causal association with dental caries. Organic acids produced by microbial hydrolysis of dietary sugars cause demineralization of hydroxyapatite crystals leading to dental caries. The Human Microbe Identification Microarray (HOMIM) for identification of named and unnamed taxa associated with oral infections [1], in combination with pyrosequencing, has enabled the complex uncultured microbial ecosystem of the oral cavity to be more precisely defined [2]–[5]. Other techniques such as scanning electron microscopy (SEM), fluorescence *in-situ* hybridization (FISH) and confocal microscopy using fluorochrome-labelled 16S rRNA probes, have given insights into the community structure and spatial distribution of polymicrobial consortia in infected tissues [6], [7]. More recently, these analyses have provided evidence indicating that a restricted group of lactobacilli, particularly *Lactobacillus rhamnosus*, are implicated in the initial stages of infection of vital pulp tissue [6].

While many current techniques have allowed partial profiling of the human microbiome, important questions regarding functional attributes, metabolic specialization and virulence traits can only be answered by comprehensive post-genomic analysis of the ecosystems of the human body [8]. Current high-throughput Next Generation Sequencing has the power to provide detailed knowledge of the diverse array of genes that influence biochemical and physiological functions responsible for host-microbe associations. This enables the identification of bacterial traits that contribute to adhesion, invasion and evasion of host defences. In the present study, whole genome sequencing of two clinical isolates of *L. rhamnosus* using Roche GS FLX+ technology combined with detailed genome-wide analysis of the genetic profiles of tissue invasive *L. rhamnosus* compared with probiotic *L. rhamnosus*, was undertaken. The hypothesis to be tested was that invasive *L. rhamnosus* possess a unique complement of genes that could facilitate invasion of dental pulp tissue.

We have generated draft genome sequences of two clinical isolates of *L. rhamnosus* strains, LRHMDP2 and LRHMDP3 [9] isolated from infected dental pulps from carious teeth. These represent the initial stages of infection of pulp tissue. Here we report comprehensive comparative genomic analysis of our two clinical isolates of *L. rhamnosus*, LRHMDP2 and LRHMDP3 (isolated from two different subjects) with probiotic *L. rhamnosus* GG (ATCC 53103) and explore the relevance of the genomic features of the clinical isolates to invasion of dental pulp. Both clinical isolates showed absence of typical exopolysaccharide (*eps*) and *Spa*CBA pilus clusters. Newly identified genes from the clinical isolates included a modified *eps* cluster, a two-component sensor kinase, a response regulator and ABC transporters for ferric iron. Additional genes detected in the clinical isolates included putative open reading frames encoding RNA polymerase sigma 54 factor RpoN, NtrC, MutR, ArsR and zinc-binding Cro/CI family transcriptional regulators. PTS system mannose, galactitol, mannitol, cellobiose and beta-glucoside-specific II components were also identified. This analysis provided evidence that clinical isolates of invasive *L. rhamnosus* differ from probiotic reference strains of this organism.

## Results and Discussion

### Genome sequencing

*L. rhamnosus* clinical isolates (LRHMDP2 and LRHMDP3) were sourced from infected dental pulps from carious teeth categorised as representing the initial stages of infection of vital pulp tissue from two different subjects [6] (see Methods section for detail description for selection criteria; **Tables S1, S2a, S2b**). These isolates were speciated by PCR analysis of 16S rRNA gene sequences using *Lactobacillus* genus-specific primers as described in an earlier report [10]. LRHMDP2 and LRHMDP3 16S rRNA gene sequences were 99% homologous to *L. rhamnosus*. Genomic DNA from both the clinical isolates was sequenced by a whole genome shotgun strategy using Roche GS FLX+ pyrosequencing at the Ramaciotti Centre for Gene Function Analysis, University of New South Wales (see Methods section for detailed sequencing protocol).

### Genome properties

**Figure 1:**
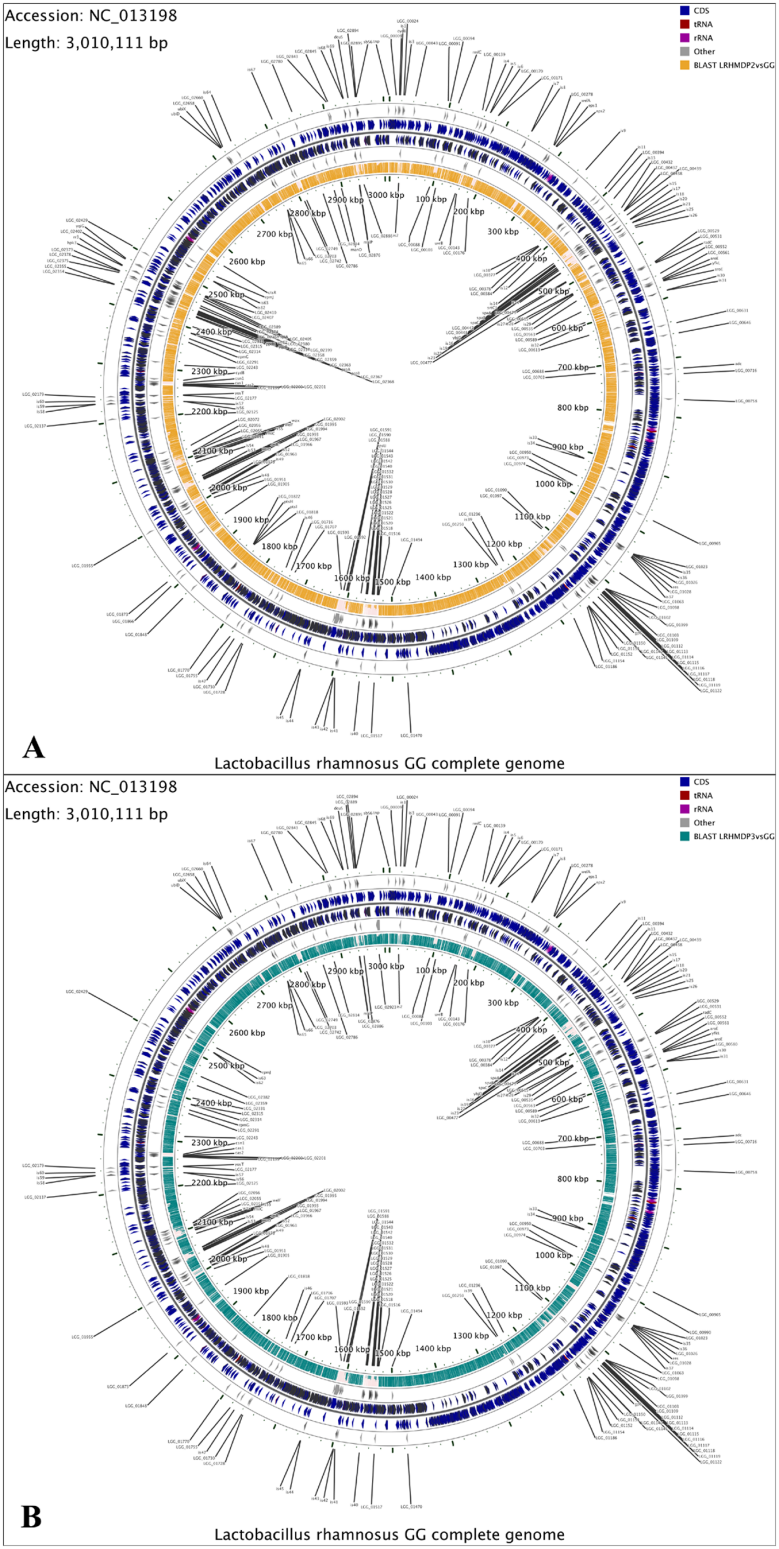
(A) Difference in gene content between *L. rhamnosus* GG and *L. rhamnosus* LRHMDP2; (B) Difference in gene content between *L. rhamnosus* GG and *L. rhamnosus* LRHMDP3. In both figures, genes from positive and negative strands of *L. rhamnosus* GG are shown in rings II and III (from outside). Protein coding genes, tRNA and rRNA genes are shown by different colours. Directions of the arrows indicate the translational directions. Genes found to be absent in *L.rhamnosus* LRHMDP2/LRHMDP3 are highlighted in rings I and IV, and are labelled using gene symbols. The innermost rings (V) represent the homology of *L.rhamnosus* LRHMDP2/LRHMPD3 against *L. rhamnosus* GG, regions. Gaps indicate lower BLAST scores between the genomes of *L. rhamnosus* LRHMDP2/LRHMDP3 and *L. rhamnosus* GG [48].

A total of 77,329 reads from *L. rhamnosus* LRHMDP2 and 75,985 reads from *L. rhamnosus* LRHMDP3 were generated to reach a depth of ∼17-fold genome coverage, and assembled by Newbler Assembler 2.6 into 51 and 47 contigs longer than 200 base pairs, respectively (**Table 1**, [9]). Gene definition and annotation was performed by merging the result from the RAST (Rapid Annotation using Subsystem Technology) server and tRNAscan-SE. This showed that sequences of the two strains, *L. rhamnosus* LRHMDP2 and *L. rhamnosus* LRHMDP3, included 2,911,290 base pairs and 2,911,934 base pairs, respectively. The genomic sequence of *L. rhamnosus* LRHMDP2 comprised 2,910 coding sequences (CDSs), 7 rRNA loci and 56 tRNAs. In comparison, the genome sequence of *L. rhamnosus* LRHMDP3 contained 2,925 CDSs, 6 rRNA loci and 57 tRNAs. The G+C content of both clinical strains of *L. rhamnosus*, LRHMDP2 and LRHMDP3 was 46.6%, similar to *L. rhamnosus* GG and *L. rhamnosus* Lc705 (**Table 1** **,** **Figure 1** [9]). By pair-wise genome comparisons Lukjancenko *et al*. have reported 93.3% similarity between two *L. rhamnosus* strains [11].

**Table 1:** General Genome sequencing^a^ features and annotation^b^ features of *L. rhamnosus* clinical isolates and *L. rhamnosus* GG and *L. rhamnosus* Lc705 genomic features.

Organism	Total Reads	genome coverage	Number of contigs^c^ (>200 bp)	Genome size (Mbp)	Coding sequences (CDS)	rRNA operons	tRNA genes	GC content (%)	Hypothetical proteins (total)
LRHMDP2^d^	77,329	17 X	51	2.91	2,910	7	56	46.6	815
LRHMDP3 d	75,985	17 X	47	2.91	2,925	6	57	46.6	819
GG^e^	-	-	-	3.01	2944	5	57	47	734
Lc705^e^	-	-	-	3.03	2992	5	61	47	760

a Using Roche GS(FLX+) pyrosequencing.

b Based on results merged from the RAST (Rapid Annotation using Subsystem Technology) server and tRNAscan-SE.

c assembled by Newbler Assembler 2.6.

d Both *L.rhamnosus* clinical isolates (LRHMDP2 and LRHMDP3) were sourced from infected dental pulps from carious teeth categorised as representing the initial stages of infection of pulp tissue.

e [13].

### Comparative genome analysis

Genome annotation using the RAST server assigns functions to genes, enables assembly of metabolic pathways and makes connections into sub-systems represented in the genome to achieve comparative analysis with annotated genomes maintained in the SEED (http://www.theseed.org/) [12]. Biological roles could be assigned to 72.0% of CDSs for the genomic sequences of *L. rhamnosus* LRHMDP2 and *L. rhamnosus* LRHMDP3. A comparative annotation summary indicated 264 of the 2,910 coding sequences from *L. rhamnosus* strain LRHMDP2 and 258 of the 2,925 coding sequences from *L. rhamnosus* strain LRHMDP3 (∼9% of coding sequences) in the clinical isolates were newly identified genes (**Table 1** **,** **Table 2**) where homologues could not be detected in the probiotic reference strain of *L. rhamnosus* ATCC 53103 (GG). New genes are defined as no BLAST hits found or the normalised BLAST score less than 2 where normalised BLAST score is calculated as BLAST score divided by alignment length. Some 9% of coding sequences attributed to genes from the probiotic *L. rhamnosus* strain GG as well as the other probiotic *L. rhamnosus* strain Lc705 were not detected in either clinical isolate (**Table 3**).

**Table 2:** Number of new genes identified in the assemblies of clinical isolates, *L. rhamnosus* LRHMDP2 and *L. rhamnosus* LRHMDP3^a^.

Assembly Name	Number of genes
*L. rhamnosus* LRHMDP2	264^b^
*L. rhamnosus* LRHMDP3	258^c^

a based on comparative studies with reference probiotic *L. rhamnosus* strains ATCC53103 (GG) and Lc705.

b inclusive of 176 genes coding for hypothetical proteins.

c inclusive of 166 genes coding for hypothetical proteins.

**Table 3:** Number of genes from reference strain *L. rhamnosus* ATCC 53103 (GG) and *L. rhamnosus* Lc705 not detected in both assemblies^a^.

Reference probiotic *L. rhamnosus* strains	Number of genes
*L. rhamnosus* ATCC 53103 (GG)	275^b^
*L. rhamnosus* Lc705	331^c^

a L. rhamnosus LRHMDP2 and L. rhamnosus LRHMDP3.

b inclusive of 77 genes coding for hypothetical proteins.

c inclusive of 106 genes coding for hypothetical proteins and 63 plasmid genes.

Comparisons based on sub-system category distribution highlighted distinctive features that discriminated the two clinical isolates of *L. rhamnosus*, (LRHMDP2 and LRHMDP3) and *L. rhamnosus* GG. Comparative genomic analysis between *L. rhamnosus* GG and the two strains, *L. rhamnosus* LRHMDP2 and *L. rhamnosus* LRHMDP3, based on 27 sub-system category distributions, showed differences in 6 distribution categories, namely; co-factors, vitamins, prosthetic groups and pigments; cell wall and capsule; virulence, disease and defence traits; phage, prophage, transposable elements, plasmids, iron acquisition and metabolism and carbohydrates (data not shown).

Compared to the *L. rhamnosus* GG genome, components not detected in either of the clinical isolate assemblies were mainly assigned to genes encoding transposases (IS5, IS30, IS3/IS911, IS150/IS3, IS4 family proteins) and phage-related products. Also, not detected in the clinical isolates were characteristic exopolysaccharide (*eps*) cluster genes, pilus-specific genes (the *spa*CBA cluster), mannose/fructose/sorbose- specific PTS system genes and genes encoding MerR and XRE family transcriptional regulators. Further omissions included type III restriction modification systems and CRISPRs and CRISPR-associated (*cas*) genes, in addition to genes coding for hypothetical proteins (**Table S3**). Similarly, genes present in the other *L. rhamnosus* probiotic strain, *L. rhamnosus* Lc705 that were absent in both clinical isolate assemblies were mainly assigned to transposases (IS111A, IS1328, IS1533, IS116, IS110, IS902, IS4, IS1480, IS30, IS5, tnp4), phage-related products, exopolysaccharide (*eps*) clusters; also fructose-specific, sorbose-specific, galactitol-specific, lactose/cellobiose-specific and mannose-specific PTS systems. Also noted as absent from the clinical isolates were other carbohydrate metabolism genes and the type I restriction modification system in addition to genes encoding hypothetical proteins (**Table S4**).

The clinical isolates, *L. rhamnosus* LRHMDP2 and *L. rhamnosus* LRHMDP3 were also distinct from the other *L. rhamnosus* probiotic strain Lc705 as no plasmid genes could be identified in either as compared to *L. rhamnosus* Lc705 that harbours a 64.5 kb plasmid [13]. However, the two clinical isolates from infected dental pulp resembled *L. rhamnosus* Lc705 and other lactobacilli such as *L. gasseri*, *L. johnsonii* and *L. iners* in the absence of CRISPRs and *cas* detected in *L. rhamnosus* GG [13], [14]. CRISPRs and *cas* comprise a distinct defence system present in ∼47% bacterial genomes (http://crispr.u-psud.fr/crispr/). This system provides resistance against foreign genetic elements and bacteriophage predation by inactivating incoming DNA. Similarly, both clinical isolates resembled *L. rhamnosus* Lc705 in the absence of the *spa*CBA cluster but presence of a homologous *spa*FED cluster also present in *L. rhamnosus* GG. In Gram-positive bacteria the three subunits of the *spa*CBA gene cluster are flanked by transposable elements and in *L. rhamnosus* strain GG this cluster is located on a genomic island GGISL2 [13]. Absence of the *spa*CBA gene cluster in the clinical isolates could be related to the paucity in these strains of transposable elements that regulate horizontal gene transfer.

Both clinical isolates were characterised by a modified *eps* cluster, a two-component sensor kinase, a response regulator and ABC transporters for ferric iron, RNA polymerase sigma 54 factor RpoN, MutR, NtrC, ArsR and zinc-binding Cro/CI family transcriptional regulators, ThiJ/PfpI family protein and PTS system mannose, galactitol, mannitol, cellobiose and beta-glucoside-specific II components in addition to genes encoding hypothetical proteins (**Table S5a, S5b**). The majority of the coding sequences for newly identified genes had closest homology with genes in bacteria from the phylum *Firmicutes*.

### Inter-strain divergence between *L. rhamnosus* strains LRHMDP2 and LRHMDP3

Although the probiotic *L. rhamnosus* strains GG and Lc705 were shown to exhibit 331 and 383 strain-specific genes respectively [13], the inter-strain difference between clinical isolates *L. rhamnosus* LRHMDP2 and *L. rhamnosus* LRHMDP3 was limited. Analysis revealed 26 strain-specific genes for *L. rhamnosus* LRHMDP2 and 28 strain-specific genes for *L. rhamnosus* LRHMDP3 (**Table S6a, S6b**). Many of the strain-specific genes encoded hypothetical proteins. Biological function could be assigned to 4 of these genes from *L. rhamnosus* LRHMDP2 and 8 from *L. rhamnosus* LRHMDP3. Type II secretory pathway/competence component/competence protein ComGC, immunity protein PlnI, membrane-bound protease CAAX family and adhesion exoprotein were unique to *L. rhamnosus* LRHMDP2. DinG family ATP-dependent helicase YoaA, a putative regulator of the mannose operon ManO, cytochrome d ubiquinol oxidase subunit I, phage lysin, flagellar hook-length control protein FliK, phosphotransferase system, phosphocarrier protein HPr, phosphoenolpyruvate-protein phosphotransferase of PTS system and transcriptional regulator CtsR, were identified only in *L. rhamnosus* LRHMDP3. Genome sequencing has identified CtsR (class III stress gene repressor) in other *L. rhamnosus* strains. However, CtsR is well characterised in *Lactobacillus plantarum* - found in vegetable, meat and dairy products and also as a natural inhabitant of the mammalian gut. CtsR plays an important role in the response to environmental stress conditions [15]. CtsR is also considered to play an important role in stress resistance and virulence in *Listeria monocytogenes*, a firmicute that causes food-borne illness and is associated with invasive gastrointestinal infection [16].

### Genomic distance and SNP divergence

The genomic difference between the genomic sequences of two *L. rhamnosus* clinical isolates and eleven other *L. rhamnosus* strains from NCBI was analysed. **Figure S1** shows a dendrogram that clusters the 13 different *L. rhamnosus* strains based on full genome nucleotide-level comparisons. The branches of the dendrogram are scaled to show the distance between genomes. The two clinical isolates show small genomic difference, and are found in a different part of the dendogram compared to the other probiotic *L. rhamnosus* dairy isolates, intestinal isolates from healthy humans and isolates from feces of healthy humans.

A more specific SNP divergence analysis between the probiotic *L. rhamnosus* strain GG and the two *L. rhamnosus* clinical isolates identified 50,459 SNPs with 95% confidence (**Table S7**). Of these, 6,899 SNPs were found to be located in non-coding regions. Both clinical isolates exhibited 50,370 SNPs in common when compared with GG. Only 30 SNPs were common between *L. rhamnosus* GG and *L. rhamnosus* LRHMDP2 compared to *L. rhamnosus* LRHMDP3. *L. rhamnosus* GG and *L. rhamnosus* LRHMDP3 shared only 45 SNPs compared to *L. rhamnosus* LRHMDP2. These findings indicate that SNP divergence between the two clinical isolates is 0.2%. The divergence equivalent to 50,459 SNPs between the probiotic *L. rhamnosus* strain GG and the two *L. rhamnosus* clinical isolates, confirmed the segregation of the clinical isolates.

### Exopolysaccharide cluster

Most of the lactic acid bacteria (LAB) produce exopolysaccharide (EPS) and the composition of exopolysaccharide has been shown to influence adhesion properties of probiotic strains [17], [18]. Metabolic shunting away from glycolysis is considered to be an important driver of EPS formation [19]. The organization of an exopolysaccharide (*eps*) gene cluster from LAB includes genes encoding glycosyltransferases (key enzymes for biosynthesis of the EPS repeating unit located in the central region of the *eps* cluster), polysaccharide biosynthesis export protein and transcriptional regulator, in addition to chain length determinant protein (*wzd* ORF) at one end of the *eps* gene cluster and protein-tyrosine phosphatase (*wzb* ORF) on the other end of the *eps* gene cluster [17], [19], [20]. To date, the *eps* cluster genes of only two probiotic *L. rhamnosus* strains GG and ATCC 9595 have been characterised in detail [18], [21].

**Figure 2:**
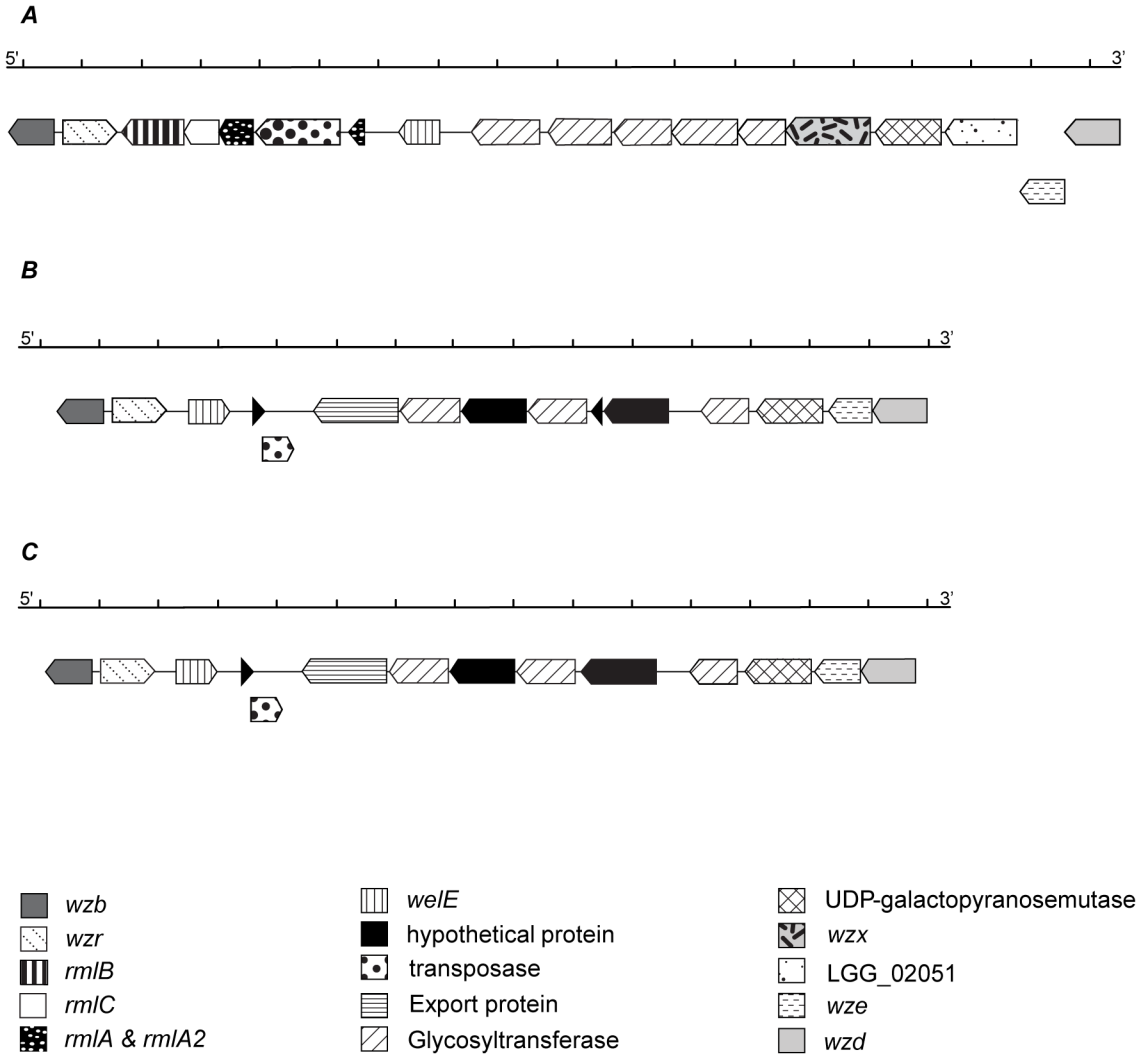
Genomic organisation of EPS gene cluster of *L. rhamnosus* GG (A), *L. rhamnosus* LRHMDP2 (B) and *L. rhamnosus* LRHMDP3 (C). Nomenclature analogy of exopolysaccharide/capsular gene cluster is maintained in accordance to *L rhamnosus* ATCC53103/GG and ATCC 9595 *eps* cluster [18], [21].

The *eps* cluster from the two clinical isolates, *L. rhamnosus* LRHMDP2 and *L. rhamnosus* LRHMDP3, is composed of 15 ORFs (12.44 kb) and 14 ORFs (11.55 kb) respectively, with notable absence of genes (*rmlACBD*) involved in dTDP-rhamnose biosynthesis (**Figure 2** **,** **Table 4** **,** **Table 5**). Genes involved in dTDP-rhamnose biosynthesis were identified in the 16.37 kb *eps* gene cluster of *L. rhamnosus* GG consisting of 17 putative ORFs [18] and the 18.7 kb *eps* gene cluster of *L. rhamnosus* ATCC 9595 consisting of 18 putative ORFs [21]. However, insertion of the putative transposase DDE domain from *L. casei* ATCC 334 (62% identity) was found to inactivate *rmlA* from the *eps* cluster of *L. rhamnosus* GG [18]. In both clinical isolates, a degenerate transposase belonging to the TnpB_IS66 superfamily and with 97% identity with the homolog from *L. rhamnosus* LMS2-1 was identified at a similar location within the *eps* cluster (**Table 4** **,** **Table 5**). The absence of *rmlACBD* genes involved in the dTDP-rhamnose biosynthetic enzyme system in the *eps* cluster from both clinical isolates implies an alternate route for EPS synthesis employed by the clinical isolates as compared to *L. rhamnosus* GG and *L. rhamnosus* ATCC 9595 [19]. The *eps* cluster of both clinical isolates also comprises 3 glycosyltranferases as opposed to 5 glycosyltranferases in *L. rhamnosus* GG [18] and in *L. rhamnosus* ATCC 9595 [21]. The presence of family 2 glycosyltransferases showing 50% amino acid identity to a homolog from *Lactobacillus pentosus* strain IG1 suggests a different pathway for polymerisation of monosaccharide repeating units. Family 2 glycosyltransferases are inverting glycosyltransferases, implying probable β-glycosidic linkage in the repeating unit of EPS (www.cazy.org/glycosyltransferases.html). A near-identical EPS biosynthetic gene cluster in *L. rhamnosus* strains ATCC 9595 and *L. rhamnosus* Lc705 produces rhamnose-rich EPS as opposed to the galactose-rich EPS from *L. rhamnosus* GG. On a broader scale, however, the organisation of the *eps* gene cluster from all the *L. rhamnosus* strains studied including the two clinical isolates, remains in close agreement. For instance, the *wzb* ORF as part of the *eps* cluster from *L. rhamnosus* strain GG and both clinical isolates are predicted to share 100% amino acid sequence identity. Remaining genes of the *eps* cluster from both clinical isolates showed closest identity to two *eps* cluster genes from *L. rhamnosus* strain LMS2-1, *Lactobacillus zeae* strain KCTC 3804 and *Lactobacillus pentosus* strain IG1 and a single *eps* cluster gene each from *L. rhamnosus* strains GG, Lc705 and HN001. Biological function is unknown for four ORFs from the *L. rhamnosus* LRHMDP2 *eps* gene cluster and 3 ORFs from the *L. rhamnosus* LRHMDP3 *eps* gene cluster whereas in *L. rhamnosus* strains GG, Lc705 and ATCC 9595, biological function could be attributed to most of the predicted translated products.

**Table 4:** Exopolysaccharide cluster genes from *L*. *rhamnosus* LRHMDP2.

ORF	Size (bp)	Predicted encoded function	Predicted domain(s) present in encoded ORF	Best BLASTx hit (accession no.)		% Amino acid identity
				Protein	Organism	
*wzb*	768	Manganese-dependent protein-tyrosine phosphatase	No hit in Pfam database	Wzb (ABV54212.1)	*L. rhamnosus* GG	100
*wzr*	894	Cell envelope-associated transcriptional attenuator LytR-CpsA-Psr, subfamily F2	1 transmembrane domain and LytR_cpsA_psr domain (PF03816)	Wzr (YP_003174725.1)	*L. rhamnosus* Lc705	95
*welE*	669	Undecaprenyl-phosphate galactosephosphotransferase	Bacterial sugar transferase (PF02397)	WelE (ABS71033.1)	*L. rhamnosus* HN001	85
	198	hypothetical protein	low complexity	conserved hypothetical protein (ZP_04441887.1)	*L. rhamnosus* LMS2-1	95
	516	Degenerate transposase	TnpB_IS66 super family (PF05717)	Transposase (ZP_04441884.1)	*Lactobacillus rhamnosus* LMS2-1	97
	1425	polysaccharide biosynthesis export protein	Polysacc_synt (PF01943) and 6 transmembrane domains	Oligosaccharide translocase (YP_003352680.1)	*Lactococcus lactis* subsp. *lactis* KF147	43
	1011	Glycosyltransferase family 2	Glycosyltransferase family 2 (PF00535)	WchA (CCC15460.1)	*L. pentosus* IG1	51
	1086	hypothetical protein	Polysaccharide pyruvyltransferase (PF04230)	Polysaccharide pyruvyltransferase (YP_005047298.1)	*Clostridium clariflavum* DSM 19732	44
	978	Glycosyltransferase group 2 family protein	Glycosyltransferase family 2 (PF00535)	WelL (CCC15461.1)	*Lactobacillus pentosus* IG1	50
	210	hypothetical protein	2 transmembrane domains	Endoplasmic reticulum metallopeptidase 1 (CCH00546.1)	*Fibrellaaestuarina* BUZ 2	34
	1101	hypothetical protein	9 transmembrane domains	hypothetical protein (ZP_06987467.1)	*Bacteroides* sp. 3_1_19	25
	792	Glycosyltransferase	No hit in Pfam database	WciB (ABQ58967.1)	*Streptococcus oralis*	53
	1122	UDP-galactopyranosemutase	UDP-galactopyranosemutase(PF03275)	UDP-galactopyranose mutase (ZP_09453942.1)	*L. zeae* KCTC 3804	88
*wze*	756	Tyrosine-protein kinase EpsD	ATPase MipZ (PF09140)andCbiA (PF01656)	Wze (ZP_09453943.1)	*L. zeae* KCTC 3804	89
*wzd*	915	Tyrosine-protein kinase transmembrane modulator EpsC	Chain length determinant protein (PF02706) and 1 transmembrane domain	Wzd (ACN94846.1)	*L. rhamnosus* GG	79

**Table 5:** Exopolysaccharide cluster genes from *L*. *rhamnosus* LRHMDP3.

ORF	Size (bp)	Predicted encoded function	Predicted domain(s) present in encoded ORF	Best BLASTx hit (accession no.)		% Amino acid identity
				Protein	Organism	
*wzb*	768	Manganese-dependent protein-tyrosine phosphatase	No hit in Pfam database	Wzb (ABV54212.1)	*L. rhamnosus* GG	100
*wzr*	894	Cell envelope-associated transcriptional attenuator LytR-CpsA-Psr, subfamily F2	1 transmembrane domain and LytR_cpsA_psr domain (PF03816)	Wzr (YP_003174725.1)	*L. rhamnosus* Lc705	95
*welE*	669	Undecaprenyl-phosphate galactosephosphotransferase	Bacterial sugar transferase (PF02397)	WelE (ABS71033.1)	*L. rhamnosus* HN001	85
	198	hypothetical protein	low complexity	conserved hypothetical protein (ZP_04441887.1)	*L. rhamnosus* LMS2-1	95
	516	Degenerate transposase	TnpB_IS66 super family (PF05717)	Transposase (ZP_04441884.1)	*Lactobacillus rhamnosus* LMS2-1	97
	1425	polysaccharide biosynthesis export protein	Polysacc_synt (PF01943) and 6 transmembrane domains	Oligosaccharide translocase (YP_003352680.1)	*Lactococcus lactis* subsp. *lactis* KF147	43
	1011	Glycosyltransferase family 2	Glycosyltransferase family 2 (PF00535)	WchA (CCC15460.1)	*L. pentosus* IG1	51
	1086	hypothetical protein	Polysaccharide pyruvyltransferase (PF04230)	Polysaccharide pyruvyltransferase (YP_005047298.1)	*Clostridium clariflavum* DSM 19732	44
	981	Glycosyltransferase group 2 family protein	Glycosyltransferase family 2 (PF00535)	WelL (CCC15461.1)	*Lactobacillus pentosus* IG1	50
	1323	hypothetical protein	9 transmembrane domains	hypothetical protein (ZP_06987467.1)	*Bacteroides* sp. 3_1_19	24
	792	Glycosyltransferase	No hit in Pfam database	WciB (ABQ58967.1)	*Streptococcus oralis*	53
	1122	UDP-galactopyranosemutase	UDP-galactopyranosemutase(PF03275)	UDP-galactopyranose mutase (ZP_09453942.1)	*L. zeae* KCTC 3804	88
*wze*	756	Tyrosine-protein kinase EpsD	ATPase MipZ (PF09140)andCbiA (PF01656)	Wze (ZP_09453943.1)	*L. zeae* KCTC 3804	89
*wzd*	915	Tyrosine-protein kinase transmembrane modulator EpsC	Chain length determinant protein (PF02706) and 1 transmembrane domain	Wzd (ACN94846.1)	*L. rhamnosus* GG	79

The genomic organisation of the *eps* cluster genes from *L. rhamnosus* GG showed that, except for one ORF (*wzr*), all other ORFs of the *eps* cluster are in reverse orientation. Whereas 11 ORFs in *L. rhamnosus* LRHMDP2 and 10 ORFs in *L. rhamnosus* LRHMDP3 are in reverse orientation to the remaining ORFs of the *eps* cluster (**Figure 2**). In both clinical isolates, in addition to *wzr* (cell envelope-associated transcriptional attenuator LytR-CpsA-Psr, subfamily F2), priming glycosyltransferase, *welE* (undecaprenyl-phosphate galactosephosphotransferase) also maintained a forward direction. These findings imply that in the clinical isolates, in addition to organisation, transcriptional direction of *wzr* and *welE* could regulate expression of *eps* cluster genes.

Minor variation in structure of the polysaccharide is found to influence adherence and biofilm formation and the nature of immune responses. A deletion mutant for the priming glycosyltransferase (*welE*) of *L. rhamnosus* GG showed altered heteropolysaccharide composition with increased adherence and biofilm capacity compared with the galactose-rich EPS from wild type *L. rhamnosus* GG [18]. A homolog for the priming glycosyltransferase *welE* from *L. rhamnosus* HN001 and a homolog for *wchA* from *L. pentosus* IG1 were detected in the *eps* cluster from both clinical isolates. In *S. pneumoniae*, *wchA* encodes the initial transferase transferring glucose-1-phosphate to undecaprenol phosphate, a similar function to *WbaP*, a galactosyl-1-phosphate transferase of the *Salmonella enterica* O-antigen gene cluster that catalyzes the first step of O-antigen synthesis [22], [23].

### Phosphotransferase system (PTS) transporters, ATP-binding cassette (ABC) transporters and sugar transporters

Genome-wide studies, using microarray-based expression analysis and also signature-tagged mutagenesis (STM) have shown that, in many bacteria, catabolism of complex carbohydrates could play a crucial role in the pathogenesis of disease [24]. Lactobacilli, including probiotic lactobacilli with a broad spectrum pattern of carbohydrate utilisation, were found to encode PTS transporters as a dominant mechanism for carbohydrate transport [13], [25], [26]. In *L. rhamnosus* LRHMDP2 and *L. rhamnosus* LRHMDP3, 85 and 86 PTS transporters respectively, were identified (equivalent to ∼3% of coding sequences) (**Table 6**). Not all PTS transporters were complete (encoding for all subunits). Both clinical isolates encoded multiple clusters for cellobiose uptake and hydrolysis as observed in *L. casei* [27]. Unlike *L. rhamnosus* GG, maltose/maltodextrin metabolism in the clinical isolates could be associated with the ABC transporter, a mechanism also adopted by *L. casei* and *Lactococcus lactis* [26], [28]. The PTS for mannose-specific IIA, IIB, IIC, IID, galactitol-specific IIA, IIC, mannitol-specific IIA, lactose-specific IIB, cellobiose-specific IIB, IIC and PTS system beta-glucoside-specific IIA, IIB, IIC identified in both clinical isolates, were not homologous to the genes in the genome of *L. rhamnosus* GG (**Table S5a, S5b**).

**Table 6:** PTS, ABC and sugar transporters in *L. rhamnosus* GG, LRHMDP2, LRHMDP3.

Organism	PTS transporters	ABC transporter	sugar transporters
LRHMDP2^a^	85	69	70
LRHMDP3^a^	86	70	71
GG^b^	75	85	56

a Count from annotations.

b count from KEGG.

Sixty nine ATP-binding cassette (ABC) transporters identified in clinical isolates included multidrug transporter, antimicrobial peptide transporter, bacteriocin/lantibiotic transporter, phosphate, nitrate/sulfonate/bicarbonate, alkane sulfonates and ferric iron, zinc and manganese transporters, oligopeptide and amino acid, glutamine, proline, glycine, betain transporters, osmotically activated L-carnitine/cholin (OpuCA, CB, CC, CD) transporters, excinuclease, spermidine, putrescine transport, glycerol-3-phosphate (UgpCAE), ribose (RbsDACB), N-acetyl-D-glucosamine, maltose/maltodextrin transporters and multisugar transporters. Most of the ABC transporters were also detected in *L. rhamnosus* GG except for ferric iron, lantibiotic, alkane sulfonates, L-carnitine/cholin, ribose, N-acetyl-D-glucosamine and maltose/maltodextrin transporters. In both clinical isolates, ferric iron ABC transporters (iron-binding protein; LRHMDP2_1748, LRHMDP3_480 and permease protein; LRHMDP2_1750, LRHMDP3_478) were associated with two-component sensor kinase (LRHMDP2_1746, LRHMDP3_482) and response regulator (LRHMDP2_1747, LRHMDP3_481). Nearest homologs for ferric iron ABC transporters (iron-binding protein and permease protein), two-component sensor kinase and response regulator could be identified in *L. casei* and *L. paracasei* strains.

Genome-wide analysis has shown that virulence attributed to ABC transporters is associated with uptake of nutrients, metal ions such as iron, zinc, and manganese and cell attachment in a given physiological niche [29], [30]. However, ABC transport systems, particularly important in bacterial virulence in disease models, were detected in clinical isolates as well as probiotic *L. rhamnosus* GG [29], [30]. ABC transporters for antimicrobial peptides were detected in both clinical isolates as well as in probiotic *L. rhamnosus* GG. Resistance towards host defence molecules such as antimicrobial peptides is identified as an important virulence phenotype and considered as a target for antimicrobial therapy for infectious diseases of humans [31].

### Surface-exposed proteins

Surface-exposed proteins were identified using genome-wide analysis for predicted signal sequences; sortase motif, lipid attachment motif, choline binding motif, YSIRK signal peptide for Gram-positive cell-wall attached proteins, membrane proteins and exported proteins (**Table 7**). In *L. rhamnosus* LRHMDP2 and *L. rhamnosus* LRHMDP3, as well as in *L. rhamnosus* GG, surface proteins with YSIRK signal peptides found in other Gram-positive cell-wall attached proteins and putative choline binding motifs, were absent. However, 12 LPXTG-type cell wall surface anchor proteins could be identified in *L. rhamnosus* LRHMDP2 and 10 in *L. rhamnosus* LRHMDP3 (**Table 8**). As expected, surface-exposed pilus-specific proteins (SpaCBA) of *L. rhamnosus* GG were not detected in either clinical isolate (see comparative genomic analysis). LPXTG-type Gram-positive anchor regions with designated biological functions were identified in both clinical isolates. These included a putative cell wall surface anchor family protein with a repeating unit of a collagen binding domain, a subtilisin-like serine protease, a putative cell wall anchored protein SasC (LPXTG motif), similar to MabA, a modulator of *L. rhamnosus* GG adhesion and biofilm formation and a cell wall surface anchor family protein (internalinJ), renamed as mucus binding factor (MBF) in *L. rhamnosus* GG. Other LPXTG-type Gram-positive anchor regions are currently identified as hypothetical proteins with no assigned biological function.

**Table 7:** Surface exposed proteins in *L. rhamnosus* GG, LRHMDP2, LRHMDP3.

Surface exposed proteins	*L. rhamnosus* GG	*L. rhamnosus* LRHMDP2	*L. rhamnosus* LRHMDP3
Putative Signal Peptides^a^	253	235	179
Candidate lipoprotein signal peptides^b^	145	141	143
Putative choline-binding motifs	0	0	0
Signal peptide YSIRK for Gram-positive cell wall-attached proteins^c^	0	0	0
Membrane proteins	756	715	706
Exported proteins	98	135	136

a Identified by SignalP4.1 using a cutoff of Y score lower limit of 0.3.

b Matches to pattern {DERK}(6)-[LIVMFWSTAG](2)-[LIVMFSTAGCQ]-[AGS]-C by prosite scan:

c Identified from proteins predicted to have signal sequences by [YS]-[SA]-[IL]-[RK](2)-x(3)-G-x(2)-S.

**Table 8:** LPXTG-type Gram-positive anchor regions.

LRHMDP2:	
gi|411186888|gb|EKS54010.1|	hypothetical protein LRHMDP2_251
gi|411186173|gb|EKS53298.1|	hypothetical protein LRHMDP2_549
gi|411185004|gb|EKS52134.1|	Subtilisin-like serine protease
gi|411183640|gb|EKS50777.1|	hypothetical protein LRHMDP2_1916
gi|411183327|gb|EKS50466.1|	internalin J
gi|411182566|gb|EKS49713.1|	Adhesion exoprotein
gi|411182429|gb|EKS49578.1|	hypothetical protein LRHMDP2_2346
gi|411181795|gb|EKS48956.1|	putative cell-wall-anchored protein SasC (LPXTG motif)
gi|411181505|gb|EKS48676.1|	Phage tail fiber protein
gi|411181513|gb|EKS48684.1|	hypothetical protein LRHMDP2_2752
gi|411181406|gb|EKS48582.1|	hypothetical protein LRHMDP2_2786
gi|411181363|gb|EKS48543.1|	Cell wall surface anchor family protein putative

LRHMDP3:	
gi|411185129|gb|EKS52258.1|	hypothetical protein LRHMDP3_629
gi|411184803|gb|EKS51934.1|	hypothetical protein LRHMDP3_1001
gi|411184385|gb|EKS51518.1|	Subtilisin-like serine protease
gi|411182972|gb|EKS50114.1|	cell surface protein
gi|411182504|gb|EKS49652.1|	hypothetical protein LRHMDP3_2273
gi|411182229|gb|EKS49382.1|	hypothetical protein LRHMDP3_2400
gi|411181925|gb|EKS49083.1|	putative cell-wall-anchored protein SasC (LPXTG motif)
gi|411181547|gb|EKS48716.1|	cell wall surface anchor family protein
gi|411181369|gb|EKS48548.1|	hypothetical protein LRHMDP3_2824
gi|411181377|gb|EKS48556.1|	Phage tail fiber protein
gi|411181289|gb|EKS48474.1|	Cell wall surface anchor family protein putative

The putative cell wall surface anchor family protein with a repeat unit of collagen binding protein domain B is unique to clinical isolates and no homolog could be identified in *L. rhamnosus* GG. Collagen binding domains (CBD) from the bacterial adhesion domain superfamily are commonly detected in Firmicutes [32]. Collagen is one of the most common components of the extracellular matrix (ECM) and binding to ECM frequently precedes the invasion of host tissues. Organic material in dentinal matrix mainly consists of collagen type I fibrils and it has been suggested that adhesion to collagen components within dentinal tubules assists bacterial invasion [33]. In earlier studies we elucidated the microbiome invading dentinal tubules *in situ* [6]. Based on the phases of infection, we could identify lactobacilli consistently in the limited and established stages of infection of dental pulp [6]. Persistence of *Enterococcus faecalis* in root canals of failed endodontic treatment was associated with collagen binding protein and serine protease activity [34]. Similarly, in *S. mutans*, a collagen-binding protein, Cnm, is implicated as a virulence factor that could contribute to infection [35]. Growing evidence for association between Microbial Surface Component Recognizing Adhesive Matrix Molecules (MSCRAMM) and disease processes implies that a cell wall surface anchor family protein with repeat units of a collagen binding domain and subtilisin-like serine protease activity could play a crucial role in the apparent invasive behaviour of *L. rhamnosus*, which could be confirmed by *in-situ* mRNA expression studies in the context of tissue patho-physiology. A subtilisin-like serine protease identified in both clinical isolates was not detected in *L. rhamnosus* GG, but is present in *L. rhamnosus* strain Lc705 [13].

In both clinical isolates the putative cell wall anchored protein SasC (LPXTG motif), a 200 amino acid Gram-positive anchor (LRHMDP2_2569 and LRHMDP3_2533), showed 97% identity to the LPXTG-motif cell wall anchor domain of the extracellular matrix protein, MabA (LGG_01865) from *L. rhamnosus* GG. However, in both clinical isolates, except for the LPXTG motif, no DUF1542 (domain of unknown function 1542) could be identified. MabA containing LPXTG-motif cell wall anchor domain with multiple DUF1542 repeat domains, was characterised as a modulator of adhesion and biofilm formation in probiotic *L. rhamnosus* GG [36]. In *S. aureus*, SasC comprises an N-terminal signal peptide with a YSIRK motif, a C-terminal LPXTG motif and a FIVAR (found in various architectures) motif that includes 17 DUF1542 repeat domains. SasC is identified as a novel factor involved in cell aggregation and biofilm formation, and is implicated in *S. aureus* colonization during infection [37]. The putative cell wall anchored protein in *L. rhamnosus* GG is devoid of the saccharide-binding FIVAR domain that mediates hyaluronate and fibronectin binding [36], [38]. In both clinical isolates, a putative homolog of the cell-wall-anchored protein SasC (LPXTG Motif) locus, LRHMDP2_2895 (comprising 1716 amino acids) and LRHMDP3_2912 (comprising 1744 amino acids) with no putative conserved domains, was also detected, showing 96% identity to LGG_01865.

Altered domain structure of the cell wall anchored proteins in both clinical isolates and in probiotic GG due to the absence of YSIRK signal peptide surface proteins, as well as absence of DUF1542 repeat domains in clinical isolates, requires further investigation.

The cell wall surface anchor family protein (internalinJ) identified in both clinical isolates showed 98% sequence identity to internalin J (LGG_02337) from *L. rhamnosus* GG. This protein was characterised and renamed as mucus binding factor (MBF) [39]. MBF, together with MabA protein, is postulated to provide ancillary support in the adhesion process once *Spa*C has established primary contact with host cells. In *E. faecalis* on the other hand, internalin was shown to confer invasiveness via a “zipper” mechanism similar to that observed during the internalin-E-cadherin-mediated entry of *L*. *monocytogenes* [40]. A *Spa*CBA cluster could not be detected in either clinical isolate; however, internalin J could have pathogenic attributes.

Progression of caries is associated with changes in nutrient availability from carbohydrate rich to a nutrient limiting tissue environment. Of note, response regulators, RpoN, NtrC, MutR and zinc-binding Cro/CI were among the newly identified genes in both clinical isolates. Of interest was RpoN (σ 54 RNA polymerase), whose nearest homolog (showing ∼86%–89% identity) was in *L. casei*. In *L. casei*, *L. plantarum* and *L. monocytogenes*, mannose PTS is controlled by σ 54 RNA polymerase [41]. In addition, ferric iron ABC transporters (iron-binding protein and permease protein) associated with two-component sensor kinase and response regulator, are unique to both clinical isolates. Positive and negative transcriptional regulators as well as two-component regulatory systems, have a significant role in the virulence and pathogenicity of the Gram-positive pathogen, *L*. *monocytogenes*. Although the aetiology of acute infection differs from chronic infection, virulence/pathogenicity islands recognised in pathogenic bacteria associated with acute infections could not be identified in either clinical isolate within the confidence limits of the draft genomes. Pathogenic Gram-positive bacteria involved in infectious diseases invariably express exopolysaccharide (or capsule), pili, PTS transporters, ABC transporters and surface-exposed proteins with adhesive properties that contribute to virulence. Paradoxically, in addition to the presence of five genomic islands, the health benefit conferred by probiotic *L. rhamnosus* GG is attributed to exopolysaccharide and pilus protein encoded by the *Spa*CBA pilus cluster that is considered to establish primary contact in adhesive processes. This mechanism is supported by ancillary involvement of MBF and MabA proteins for prolonged persistence of *L. rhamnosus* GG in the gastrointestinal tract.

Comparative genomics based on COG analysis of bifidobacteria, lactobacilli and other probiotic bacteria has also indicated that virulence genes are not a separate category, but instead pathogenic strains are characterised by variation in genes assigned to COG categories M (cell wall/membrane biosynthesis) and O (post-translational modifications and chaperones) [11]. Cai *et al*. proposed a model for niche-associated evolution in *L. casei* showing evolution of *L. casei* niche generalists from the *Lactobacillus* ancestor with gain and/or loss of plasmids and genomic islands. They have also proposed that enhanced fitness of dairy specialist *L. casei* occurred by further gene loss from niche generalists capable of adaptation to wider range of environmental conditions [42]. Genetic divergence between *L. rhamnosus* clinical isolates implicated in infection of dental pulp and probiotic *L. rhamnosus* GG provides an indication of possible niche adaptation. This is a phenomenon prevalent in the evolution of lactobacilli associated with mucosal surfaces and also from food-related habitats [43], [44].

## Conclusions

Dental caries is a progressive chronic infection associated with polymicrobial aetiology. Progression of lactobacilli through dentinal tubules and the presence of apparently encapsulated *L. rhamnosus* in the initial stages of pulp infection are indicative of an important role in advanced stages of the infection. In both clinical isolates, the absence of the *L. rhamnosus* GG *Spa*CBA pilus cluster (**Table S9**), existence of a modified MabA-like protein, a unique repeat unit of a collagen binding protein domain and the presence of an altered exopolysaccharide gene cluster (**Table 4** **,** **Table 5** **, Table S9**), suggests the surface components of the clinical isolates of *L. rhamnosus* may employ a different mechanism for invasion of dental pulp. Surface components of *L. rhamnosus* clinical isolates are postulated to facilitate the observed invasion of dental pulp by adherence both to cellular elements and extracellular matrices. Invasion could also be facilitated by the evasion of host defence mechanisms, through the masking effect of an extracellular polysaccharide layer. The presence of capsular polysaccharide (CPS) or extracellular polysaccharide (EPS) is wide-spread in Gram-positive and Gram-negative pathogens. These serve dual roles in survival and pathogenic action by modulating adaptive immune responses and by regulating phagocytosis [45]. Therefore, transcriptional orientation of the *eps* cluster genes, the presence of two genes homologous to priming glycosytransferases, the absence of *rmlACBD* genes involved in the dTDP-rhamnose biosynthetic pathway and the presence of a family 2 glycosyltransferase in the *eps* cluster of both clinical isolates of *L. rhamnosus*, is predicted to alter EPS composition and could influence pathogenicity. Interestingly, the presence of putative CDSs encoding CtsR (class III stress gene repressor), RpoN (σ54 RNA polymerase), mannose PTS and internalin J as identified in the clinical isolates, correlates with the presence of homologues in *L. monocytogenes*, a firmicute involved in invasive gastrointestinal infection.

The impact of acquisition of new genes by the two clinical isolates as evident in the segregation of the clinical isolates from other probiotic *L. rhamnosus* strains and specifically, the SNP divergence from *L. rhamnosus* GG, will be better understood with future transcriptomic and functional studies.

## Materials and Methods

### Collection and processing of carious teeth

Carious teeth were collected from adult patients attending the Westmead Centre for Oral Health. The protocol was approved by the Human Ethics Committee, Sydney West Area Health Service. The decision by the clinician, independent from the study, to extract the carious tooth, was based on clinical examination and radiographs; alternatively, extraction of the tooth was the patient's informed decision. The patient signed the consent form to participate in the research. The carious tooth was not included in the study if the patient had antibiotic administered in the two weeks preceding the extraction or if the patient had reported a history of Hepatitis C or HIV. Early stage of pulp infection was inferred based on radiographic image(s) showing integrity of periodontal ligament with no shadow effect for peri-apical infection and with no clinical signs or symptoms of irreversible pulpitis, based on patient descriptors such as thermal sensitivity, pain and postural variation. Stages of infection were subsequently re-assessed during downstream processing of the tissues. Carious teeth meeting the above criteria were transported in sterile and reduced PBS glycerol to the Institute of Dental Research within 2 h of extraction and were processed. Carious dentine was excavated to near totality and the scored tooth was rinsed in sterile 18-MΩ water twice before splitting to avoid contamination from the carious dentine. A longitudinal groove was carefully carved around the carious tooth to bisect the carious lesion using a sterile bur and pressure syringe and sterile 18-MΩ water; the tooth was split sagittaly with sterile hand instruments and the extent and location of the carious lesion and patho-physiology of pulp tissue was noted. Based on these observations, classification of the carious lesion for stages of infection was re-assessed. An intact pulp with mild erythema was provisionally classified as at an early (limited) stage of infection. Exposed pulp localized on one side of the bisected tooth was carefully lifted with sterile tweezers, rinsed in sterile, reduced PBS with 15% glycerol and placed in a sterile screw capped cryo-tube containing 0.4 ml sterile PBS with 15% glycerol. The resuspended pulp was homogenized using a 2 ml sterile glass homogenizer and stored at −80°C after subsequent processing.

### PCR analysis of bacterial phyla in infected pulp tissue

Earlier findings based on FISH studies [6] indicating that fewer taxa are detected in early stages of infection was confirmed by PCR analysis of bacterial phyla in infected pulp tissue (**Table S1**).

### Isolation and identification of lactobacilli from infected vital pulp

An aliquot (20 μl) of homogenized pulp sample was streaked on freshly prepared Man, Rogosa and Sharpe (MRS) agar and incubated anaerobically at 37°C for 48 h. Each bacterial colonial form with distinct morphology was subcultured in MRS medium (2 ml). Cultures were incubated anaerobicaly at 37°C for 24 h. An aliquot (0.85 ml) of bacterial culture was collected in screw capped cryo-tubes containing 0.15 ml sterile glycerol, homogenized and stored at −80°C for future use. Remaining culture was processed for routine Gram stain to study bacterial morphology and for purification of genomic DNA as described [6]. Briefly, an aliquot (40 μl) of homogenised pulp was digested at 56°C for 40 min in cell lysis buffer consisting of 2 mg ml^−1^ proteinase K (Qiagen), 2 mg ml^−1^lysozyme (Roche) and 1000 U ml^−1^mutanolysin (Sigma) in 10 mM sodium phosphate buffer pH 6.7. After the addition of 0.1%SDS (Sigma), DNA was extracted using QIAamp DNA Mini kit (Qiagen) to give a final elution volume of 200 μl as described [6]. Genomic DNA isolated from bacterial cultures showing morphology of Gram-positive rods was subjected to PCR using *Lactobacillus* genus-specific and species-specific primers. Qualitative PCR and sequence identity for the 16S rRNA gene was performed as described previously [10].

### Design and evaluation of PCR primers

Primers were designed using Primer Express (Applied Biosystems) for the detection of bacteria from the families, *Streptococcaceae*, *Acidaminococcaceae*, *Lachnospiraceae* and *Coriobacteriaceae*as well as from *P. alactolyticus* and *Propionibacterium* strain FMA5 (**Table S2a**). Primers used for the detection of *Lactobacillaceae*, *Prevotellaceae* and *F. nucleatum* have previously been reported (**Table S2a**). Primers for the *L. rhamnosus* exopolysaccharide and pilus cluster gene set (**Table S8**) were designed using OligoExplorer 1.2 (Gene Link) and the specificity of the primers determined by DNA sequencing of the amplicons produced (Australian Genome Research Facility).

### DNA amplification for identification of bacteria

Amplicons of 16S rRNA genes for given taxa were obtained from carious dentine or infected pulp using HotStarTaq Master Mix kit (Qiagen), using 100 nM of each of the appropriate primer set (Invitrogen or Sigma; **Table S2**) and 2 μl extracted DNA solution from the infected pulp. The PCR reaction was carried out using a GeneAmp PCR system 9700 (Applied Biosystems) in a 25 μl reaction volume at 95°C for 10 min followed by 40 cycles at 95°C for 15 s and 60°C or 62°C for 1 min or 2 min, respectively. PCR products were subjected to electrophoresis on 1.6% agarose gels containing ethidium bromide and DNA bands visualized by UV illumination.

### DNA amplification for identification of *L. rhamnosus* exopolysaccharide and pilus cluster genes

In order to amplify specific genes, PCR was performed with HotStarTaq Master Mix (Qiagen), 200 nM of the appropriate primers (Integrated DNA Technologies; **Table S8**) and 2 μl of extracted DNA from eight *L. rhamnosus* clinical isolates. Amplification was performed at 95°C for 15 min followed by 40 cycles of 95°C for 15 s and 58°C for 1 min or 55°C for 1 min or 56°C for 2 min (**Table S8**) for the detection of exopolysaccharide and pilus cluster genes. DNA isolated from *L. rhamnosus* ATCC 53103 and *L. rhamnosus* ATCC 9595 was included as a positive control. The PCR products were subjected to electrophoresis as described above.

### DNA sequencing

PCR amplicons were purified using a MO BIO kit (MO BIO Laboratories Inc.) and sequenced at the Australian Genome Research Facility using the appropriate forward primer. Sequence identity of 16S rDNA PCR amplicons was established using BLAST to search nucleotide collection (nr/nt) databases accessed through NCBI (http://www.ncbi.nlm.nih.gov/).

### Whole genome sequencing using Roche GS -FLX+

Two genomic DNA samples (1–2 μg DNA, A260/A280 ratio in the range 1.8–2.0) were delivered to the Ramaciotti Centre for Gene Function Analysis, University of New South Wales, Australia for whole genome sequencing using Roche GS FLX+. The sequencing was undertaken according to standard Roche/FLX procedures.

### Genome Assembly and BLAST analysis

The sequence reads were assembled *de novo* with Newbler assembler 2.6 using the default parameters. The contigs assembled by Newbler were then joined by CAP3 [46] using default parameters. The final sets of contigs were cross-checked against the *Lactobacillus rhamnosus* GG genome sequence using blastn as well as blastx with an E-value cut-off of 10e^−6^. The absence of a gene was defined as no BLAST hits against the reference or a normalized blastx score of less than 2, where normalized blastx score was calculated as BLAST score divided by alignment length.

### Genomic Distance Analysis and SNP Detection

Genomic distance between the eleven probiotic *L. rhamnosus* strains; GG, Lc705, HN001, R0011, LMS2-1, CASL, LOCK900, LOCK908, ATCC 8530, ATCC 21052, MTCC 5462 and the 2 clinical isolates *L. rhamnosus* LRHMDP2 and *L. rhamnosus* LRHMDP3 was analyzed by whole-genome BLAST comparison using the NCBI web-based BLAST, with an E-value threshold of 10^−6^. The whole genome sequence of *L. rhamnosus* GG was used as a query sequence in the BLAST search against the other genomes. The Tree View option was then selected to generate a dendrogram using the neighbour-joining method that clusters sequences according to their distances from the query sequence.

SNP divergence between the probiotic *L. rhamnosus* strain GG and the two clinical isolates *L. rhamnosus* LRHMDP2 and *L. rhamnosus* LRHMDP3 was investigated using the web-based program SNPs Finder (http://snpsfinder.lanl.gov) [47]. The 3 genomes were uploaded at the same time for SNP detection. SNPs were evaluated in homologous regions of 600 bp that shared sequence similarity of at least 95%. Protein coding information for *L. rhamnosus* GG was used as the gene coordinate file in SNP detection for anchoring gene positions.

## Supporting Information

Figure S1 **Dendrogram of genomic difference between the 2 clinical isolates,** ***L. rhamnosus*** **LRHMDP2 and** ***L. rhamnosus*** **LRHMDP3 and 11 other L.rhamnosus strains based on genomic BLAST.** Genomic distance between eleven *L. rhamnosus* strains; GG, Lc705, HN001, R0011, LMS2-1, CASL, LOCK900, LOCK908, ATCC 8530, ATCC 21052, MTCC 5462 and the 2 clinical isolates *L. rhamnosus* LRHMDP2 and *L. rhamnosus* LRHMDP3 was analyzed by whole-genome BLAST comparison using the NCBI web-based BLAST, with an E-value threshold of 10^−6^. The whole genome sequence of *L. rhamnosus* GG was used as a query sequence in the BLAST search against the other genomes. The dendrogram was generated using the neighbour-joining method that clusters sequences according to their distances from the query sequence. Percent relatedness is indicated on the scale.
(TIF)

Table S1 **PCR analysis of bacterial taxa in infected dental pulp tissue.**
(DOC)

Table S2 **Primers used to amplify taxa (a) and Specificity of primer pairs for detecting a given taxon (b).**
(DOC)

Table S3 **Genes from the reference** ***L. rhamnosus*** **strain GG not present in both assemblies.**
(XLS)

Table S4 **Genes from the reference** ***L. rhamnosus*** **strain Lc705 not present in both assemblies.**
(XLS)

Table S5 **New genes present in** ***L. rhamnosus*** **LRHMDP2 (a) and** ***L. rhamnosus*** **LRHMDP3 (b).**
(XLS)

Table S6 **Strain specific genes for** ***L. rhamnosus*** **LRHMDP2 (a) and** ***L. rhamnosus*** **LRHMDP3 (b).**
(XLS)

Table S7 **SNP divergence between** ***L. rhamnosus*** **GG and** ***L rhamnosus*** **LRHMDP2 and LRHMDP3.**
(XLS)

Table S8 **Primers used to amplify selected exopolysaccharide genes and pilus cluster genes.**
(DOC)

Table S9 **Targeted genetic profile of exopolysaccharideand pilus cluster genes for clinical isolates from pulp sample.**
(DOC)
