# Supplementary figures and images for: Comparative Genome Analysis of Lactobacillus rhamnosus Clinical Isolates from Initial Stages of Dental Pulp Infection: Identification of a New Exopolysaccharide Cluster
Source: PLoS One. 2014 Mar 14;9(3):e90643. doi: 10.1371/journal.pone.0090643 (PMC3954586; doi:10.1371/journal.pone.0090643)

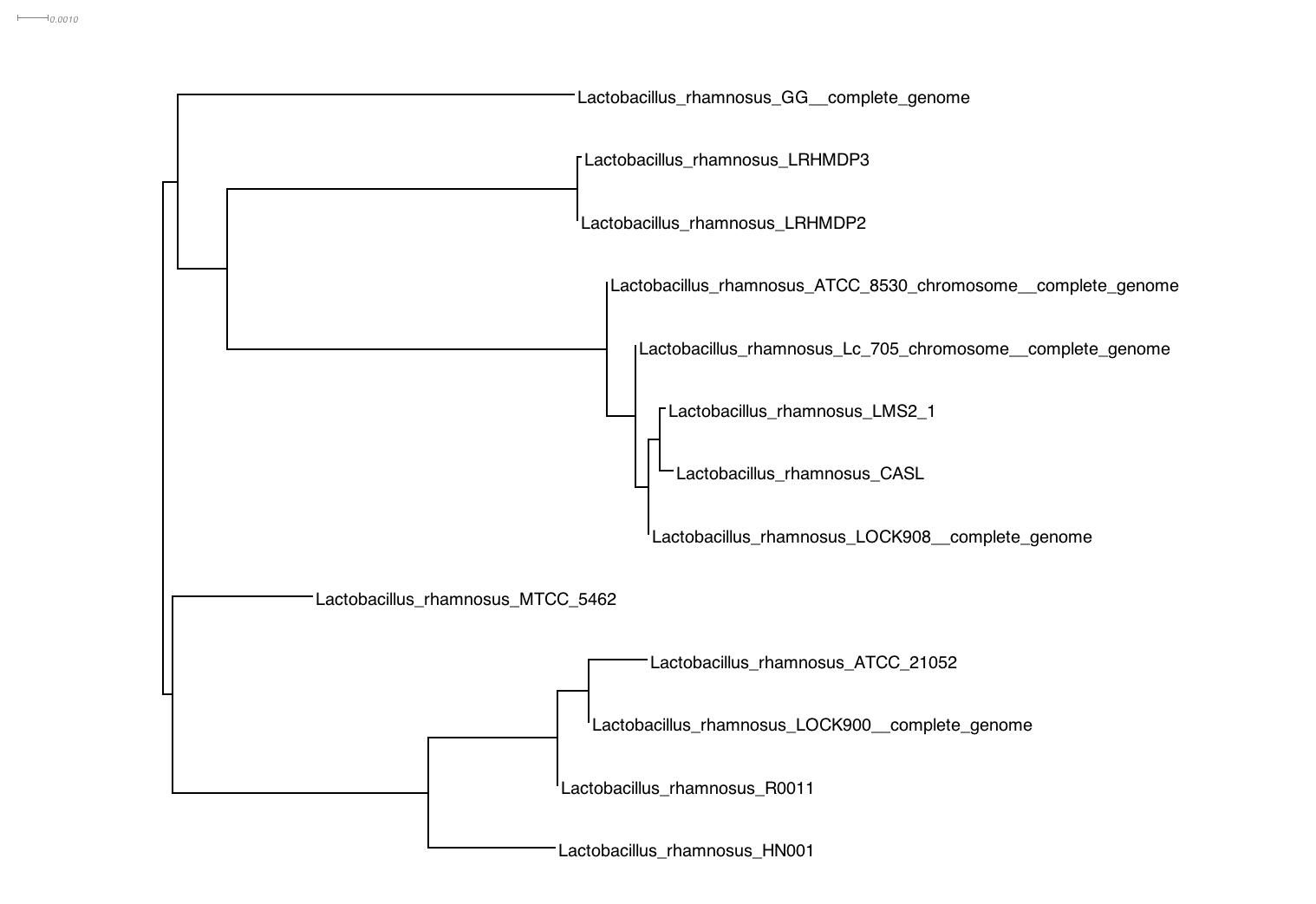

Supplement: Figure S1 — Dendrogram of genomic difference between the 2 clinical isolates, L. rhamnosus LRHMDP2 and L. rhamnosus LRHMDP3 and 11 other L.rhamnosus strains based on genomic BLAST. Genomic distance between eleven L. rhamnosus strains; GG, Lc705, HN001, R0011, LMS2-1, CASL, LOCK900, LOCK908, ATCC 8530, ATCC 21052, MTCC 5462 and the 2 clinical isolates L. rhamnosus LRHMDP2 and L. rhamnosus LRHMDP3 was analyzed by whole-genome BLAST comparison using the NCBI web-based BLAST, with an E-value threshold of 10−6. The whole genome sequence of L. rhamnosus GG was used as a query sequence in the BLAST search against the other genomes. The dendrogram was generated using the neighbour-joining method that clusters sequences according to their distances from the query sequence. Percent relatedness is indicated on the scale. (TIF) [file pone.0090643.s001.tif]
