# Supplementary material for: Comparative Genome Analysis of Lactobacillus rhamnosus Clinical Isolates from Initial Stages of Dental Pulp Infection: Identification of a New Exopolysaccharide Cluster
Source: PLoS One. 2014 Mar 14;9(3):e90643. doi: 10.1371/journal.pone.0090643 (PMC3954586; doi:10.1371/journal.pone.0090643)
Supplement: Table S1 — PCR analysis of bacterial taxa in infected dental pulp tissue. (DOC) [file pone.0090643.s002.doc]

**Table S1. PCR analysis of bacterial taxa in infected dental pulp tissue.**

| Bacteria | Pulp DNA sample | |
| --- | --- | --- |
|  | *L. rhamnosus*LRHMDP2 | *L. rhamnosus*LRHMDP3 |
| *Lactobacillaceae* | √ | √ |
| *Prevotellaceae* | ND | ND |
| *Strepcoccaceae* | ND | √ |
| *Acidaminococcaceae* | ND | √ |
| *Pseudoramibacteralactolyticus* | ND | ND |
| *Coriobacteriaceae* | ND | ND |
| *Propionibacterium*FMA5 | ND | ND |
| *Fusobacteriumnucleatum* | ND | ND |
| *Lachnobacteriaceae* | ND | ND |

√: detected; ND: Not detected
