# Supplementary material for: Comparative Genome Analysis of Lactobacillus rhamnosus Clinical Isolates from Initial Stages of Dental Pulp Infection: Identification of a New Exopolysaccharide Cluster
Source: PLoS One. 2014 Mar 14;9(3):e90643. doi: 10.1371/journal.pone.0090643 (PMC3954586; doi:10.1371/journal.pone.0090643)
Supplement: Table S2 — Primers used to amplify taxa (a) and Specificity of primer pairs for detecting a given taxon (b). (DOC) [file pone.0090643.s003.doc]

**Table S2.** **Primers used to amplify taxa (a) and specificity of primer pairs for detecting a given taxon (b).**

**a.**

| Taxon | Forward primer (5′→3′) | Reverse primer (5′→3′) | Annealing temperature and time | Amplicon length | Reference |
| --- | --- | --- | --- | --- | --- |
| Identification of bacteria | | | | | |
|  | | | | | |
| 16S rRNA gene | | | | | |
| *Lactobacillicaceae* | TGGAAACAGRTGCTAATACCG | GTCCATTGTGGAAGATTCCC | 60^o^C/1 min | 203bp | [10] |
| *Prevotellaceae* | CCAGCCAAGTAGCGTGCA | TGGACCTTCCGTATTACCGC | 62^o^C/1 min | 150bp | [49] |
| *Streptococaceae* | AGTAACGCGTAGGTAACCTGCC | TAGTTAGCCGTCCCTTTCTGGT | 62^o^C/1 min | 370bp | This study |
| *Acidaminococcaceae* | GTAGCCGGTCTGAGAGGATGA | TTATTGCGTTAACTCCGGCAC | 62^o^C/1 min | 639bp | This study |
| *P. alactolyticus* | GCCTCGAGAAATCGGGAGT | ACTGACTTATTCGCCAACACCTAGT | 60^o^C/1 min | 656bp | This study |
| *Coriobacteriaceae* | GGTTGAGAGACCGACCGG | CGTATTACCGCGGCTGCT | 60^o^C/1 min | ~300bp | This study |
| *Propionibacterium* FMA5 | TTCAGTTTCCCGGGCATC | ACTCGCGCTTCGTCATGG | 60^o^C/1 min | 281bp | This study |
| *F. nucleatum* | AAGCGCGTCTAGGTGGTTATGT | TGTAGTTCCGCTTACCTCTCCAG | 60^o^C/1 min | 106bp | [49] |
| *Lachnospiraceae* | TAGCCGATCTGAGAGGATGA | TTCCCTGCTGATAGAGCTTTACATAC | 60^o^C/1 min | 164bp | This study |

49. Martin FE, Nadkarni MA, Jacques NA, Hunter N (2002) Quantitative microbiological study of human carious dentine by culture and real-time PCR: association of anaerobes with histopathological changes in chronic pulpitis. J Clin Microbiol 40:1698-1704.

b.

| Taxon Amplified | Taxon detected | | | | | | | | |
| --- | --- | --- | --- | --- | --- | --- | --- | --- | --- |
|  | *L. acidophilus* | *P.melaninogenica* | *S. mutans* | *V. parvula* | *P. alactolyticus* | *O. profusa* | *P. acnes* | *F. nucleatum* | *L. multipara* |
| *Lactobacillaceae* | √ |  |  | √ |  |  |  |  |  |
| *Prevotellaceae ^a^* |  | √ |  |  |  |  |  |  |  |
| *Streptococcaceae^b^* |  |  | √ |  |  |  |  |  |  |
| *Acidaminococcaceae^c^* |  |  |  | √ | √ |  |  |  |  |
| *P. alactolyticus* |  |  |  |  | √ |  |  |  |  |
| *Coriobacteriaceae^d^* |  |  |  |  |  | √ |  |  |  |
| *Propionibacterium* FMA5*^e^* |  |  |  |  |  |  |  |  |  |
| *F. nucleatum* |  |  |  |  |  |  |  | √ |  |
| *Lachnospiraceae* |  |  |  |  |  |  |  |  | √ |

√: detected

*^a^* Primers for *Prevotellaceae* also detected *P. nigrescens, P. tannerae, P. denticola, P. olurum, P. intermedia, P. oris, P. buccalis, P. veroralis, P. oralis, P. shahii, P. salivae, P. buccae,P. multiformis* and *P. loeshii*.

*^b^* Primers for *Streptococcaceae* also detected *S. gordonii, S. sanguinis, S. pyogenes, S. anginosus, S. constellatus, S. mitis, S. bovis, S. equinus* and *S. salivarius*.

*^c^* Primers for *Acidaminococcaceae* also detected  *S. infelix*.

*^d^*Primers for *Coriobacteriaceae* also detected *A. rimae* and *A. parvula.*

*.^e^*Primers for *Propionibacterium* FMA5 were positive for self detection.
