# Supplementary material for: Comparative Genome Analysis of Lactobacillus rhamnosus Clinical Isolates from Initial Stages of Dental Pulp Infection: Identification of a New Exopolysaccharide Cluster
Source: PLoS One. 2014 Mar 14;9(3):e90643. doi: 10.1371/journal.pone.0090643 (PMC3954586; doi:10.1371/journal.pone.0090643)
Supplement: Table S8 — Primers used to amplify selected exopolysaccharide genes and pilus cluster genes. (DOC) [file pone.0090643.s009.doc]

**Table S8. Primers used to amplify selected exopolysaccharide genes and pilus cluster genes.**

| Gene/Locus Tag | Forward primer (5′→3′) | Reverse primer (5′→3′) | Annealing temperature and time | Amplicon length | Reference |
| --- | --- | --- | --- | --- | --- |
| Exopolysaccharide cluster genes of *L. rhamnosus* ATCC 53103 (GG), *L. rhamnosus* ATCC 9595, *L. rhamnosus* Lc705 | | | | | |
|  |  |  |  |  |  |
| LGG_Wzb_02036 | CTTGAACGCTGCACTCATCTC | CGGATTAACGGTCAGTTGTTAGA | 58^o^C/1 min | 389 bp | This study |
| LGG_Wzd­_02053 | TCAGTCAATTCACGTACAAGCC | CGTTACAGGAACACGCGAA | 58^o^C/1 min | 380 bp | This study |
| Lr9595_Wzd | CTTTAGGTTAGGAGTGACAG | GTTGCTGATGATCTCACGAG | 55^o^C/1 min | 387 bp | This study |
| LGG_Wzx_02049 | AACAGGAGATATCGAAGCGA | CCGTTATCGCGTATCTTACC | 55^o^C/1 min | 635 bp | This study |
| LGG_Wzy_02051 | TGTCTTTGGTGACACCTTCT | GCGTGGTTCGCTAATTTCAG | 55^o^C/1 min | 557 bp | This study |
| Lc705_Wzx_2048 | CTTGAACTCCAAAACTCGGT | GCTCTATGCAGTTGCTAATA | 55^o^C/1 min | 407 bp | This study |
| Lc705_Wzy_2043 | CCTGCCAGATTCAGGATAAA | CAAAGTGCATTCGGAACCTT | 55^o^C/1 min | 423 bp | This study |
|  |  |  |  |  | This study |
| Pilus cluster genes of *L. rhamnosus* ATCC 53103 (GG) | | | | | |
|  |  |  |  |  |  |
| LGG_SpaA_0442 | TCTGTATTGGCAGCAGCATC | TCTCGGGTTTAATGGCACTC | 56^o^C/2 min | 779 bp | This study |
| LGG_SpaB_0443 | CGTTTGTGGCAACAATTGAC | TCCTTCCGTCCGTTAGTGAT | 56^o^C/2 min | 611 bp | This study |
| LGG_SpaC_0444 | CTGATCAATCCCAGTGCGAT | ACCAGCAAATTCTGACGCTAAC | 56^o^C/2 min | 468 bp | This study |
| LGG_SpaD_2370 | AACAGGTTTCGTACCGCATC | CGGACGCCTTTTACCAATTA | 56^o^C/2 min | 687 bp | This study |
| LGG_SpaE_2371 | TGGCCGTCAATTAACACAAA | TATGACGCGTAAGCAAGCAC | 56^o^C/2 min | 704 bp | This study |
| LGG_SpaF_2372 | GGCCATTTTCATCAGTCGTT | CTACCGGAGCATGTCGAGTT | 56^o^C/2 min | 798 bp | This study |
