# Supplementary material for: Comparative Genome Analysis of Lactobacillus rhamnosus Clinical Isolates from Initial Stages of Dental Pulp Infection: Identification of a New Exopolysaccharide Cluster
Source: PLoS One. 2014 Mar 14;9(3):e90643. doi: 10.1371/journal.pone.0090643 (PMC3954586; doi:10.1371/journal.pone.0090643)
Supplement: Table S9 — Targeted genetic profile of exopolysaccharideand pilus cluster genes for clinical isolates from pulp sample. (DOC) [file pone.0090643.s010.doc]

**Table S9. Targeted genetic profile of exopolysaccharideand pilus cluster genes for clinical isolates from pulp sample.**

| **Clinical isolates from Pulp sample^§^** | **Exopolysaccharide (Eps) genes** | | | | **Pilus - cluster I** | | | **Pilus – cluster II** | | |
| --- | --- | --- | --- | --- | --- | --- | --- | --- | --- | --- |
|  | ***Wzb*** | *Wzd**! | *Wzx**! | *Wzy**! | *SpaC** | *SpaB** | *SpaA** | ***SpaF**** | ***SpaE**** | ***SpaD**** |
| LRHMDP2^#^ | √ | ND | ND | ND | ND | ND | ND | √ | √ | √ |
| LRHMDP21 | √ | ND | ND | ND | ND | ND | ND | √ | √ | √ |
| LRHMDP3^#^ | √ | ND | ND | ND | ND | ND | ND | √ | √ | √ |
| LRHMDP31 | √ | ND | ND | ND | ND | ND | ND | √ | √ | √ |
| LRHMDP32 | √ | ND | ND | ND | ND | ND | ND | √ | √ | √ |
| LRHMDP33 | √ | ND | ND | ND | ND | ND | ND | √ | √ | √ |
| LRHMDP34 | √ | ND | ND | ND | ND | ND | ND | √ | √ | √ |
| LRHMDP35 | √ | ND | ND | ND | ND | ND | ND | √ | √ | √ |

**^§^**Gram positive rods identified as *L. rhamnosus* with apparent extracellular capsule layer on India Ink capsule staining and anti-*L. rhamnosus* polysaccharide antibody testing [6].

^#^ Clinical isolates for Whole genome sequencing

** L. rhamnosus* GG /ATCC 53103 [18]; ! *L. rhamnosus* Lc705 /ATCC 9595 [21].

√: detected; ND: Not detected
